# Friedelin Alleviates the Pathogenesis of Collagenase-Induced Tendinopathy in Mice by Promoting the Selective Autophagic Degradation of p65

**DOI:** 10.3390/nu14081673

**Published:** 2022-04-18

**Authors:** Huaji Jiang, Xuemei Lin, Wei Liang, Yiqiang Li, Xiao Yu

**Affiliations:** 1 Department of Immunology, School of Basic Medical Sciences, Southern Medical University, Guangzhou 510515, China; gukejianghuaji@163.com; 2 Department of Orthopaedics, Yuebei People’s Hospital Affiliated to Medical College of Shantou University, Shaoguan 512026, China; beiji3feng@163.com; 3 Department of Pediatric Orthopedics, Guangzhou Women and Children’s Medical Center, Guangzhou Medical University, Guangzhou 510623, China; lynn_xm@163.com; 4 Guangdong Provincial Key Lab of Single Cell Technology and Application, Southern Medical University, Guangzhou 510515, China

**Keywords:** tendinopathy, friedelin, autophagic degradation, p65, ubiquitination

## Abstract

With the development of an aging population, tendinopathy has become a common musculoskeletal disease in the elderly with a high recurrence rate and no curative treatment. The inflammation mediated by NF-κB signaling plays an important role in tendon senescence and degeneration. Friedelin (FR) is a triterpenoid derived from green plants, which has a variety of pharmacological functions, such as analgesia, anti-inflammation, antioxidation, and anti-tumor functions. However, the role and mechanism of FR in tendinopathy are unclear. Here, we found that FR improved the mechanical strength of the Achilles tendon, restored the orderly arrangement of collagen fibers, reduced inflammatory cell infiltration, and promoted tenogenesis, thereby blocking the progression of tendinopathy. Mechanistically, FR promoted the autophagic degradation of p65 by enhancing the interaction between p62 and p65 and effectively inhibited the activation of the NF-κB pathway, thus alleviating the inflammatory response of tenocytes. In addition, FR recruited E3 ubiquitin enzyme RNF182 to increase the K48-linked ubiquitination of p65 and promoted p62-mediated autophagic degradation. Furthermore, blocking ubiquitination reversed the degradation of p65 by FR. Therefore, these findings identify the new pharmacological mechanism of the anti-inflammatory effect of FR and provide a new candidate drug for the treatment of tendinopathy.

## 1. Introduction

Tendinopathy, a degenerative disease of a tendon, accounts for about 1/3 of musculoskeletal disorders [1,2]. With the development of society and the aging of the population, the incidence of tendinopathy is increasing year by year [3,4,5]. In terms of the incidence group, tendinopathy tends to occur in the elderly [6,7], and epidemiological investigation shows that the incidence of tendon injury in the elderly is significantly higher than in the young [8,9]. Tendinopathy is common in the Achilles tendon, patellar tendon, and rotator cuff tendons [10] and often presents with localized pain and mobility impairment. Long-term chronic tendinopathy increases the risk of tendon rupture, which can lead to disability in severe cases [11,12,13]. Currently, tendinopathy is mainly treated with conservative therapy, including non-steroidal anti-inflammatory drugs, shockwave therapy, ultrasound-guided percutaneous electrolysis, and exercise rehabilitation therapy [14,15]. However, these treatments can only relieve symptoms but not stop the progression of tendinopathy. Furthermore, non-steroidal anti-inflammatory drugs have many complications, such as peptic ulcers, cardiovascular disease, and renal impairment [16]. Patients with tendon rupture or giant tendon calcification usually need surgical treatment. However, the surgical trauma is significant, and the recurrence rate is high (40%) [2], causing a heavy blow to the patient’s body and mind. Therefore, it is urgent to explore novel potential therapies for tendinopathy.

It is currently believed that tendinopathy begins after an acute reactive tendon injury, with subsequent disordered or poor repair progressing to chronic tendinopathy [17]. The etiology is complex and multifactorial, and aging is one of the important risk factors of tendinopathy [18,19], especially when the immune system undergoes complex remodeling with age, in which senescent cells are more prone to developing chronic inflammatory responses. In addition, decreased immune function in elderly patients is associated with disturbances in the gut microbiota, which may be related to pain in bone and joint diseases [20,21]. Recent studies have shown that inflammation plays a key role in the aging of tendons [22,23,24]. Inflammatory mediators (IL-1β, IL-6 and TNF-α) have been reported to be highly expressed in tendon diseases and accelerate the progression of tendinopathy [22,23]. Meanwhile, inflammatory mediators can also induce tendon fibrosis and reduce the mechanical properties of tendons [25]. In addition, inflammatory factors inhibit the tenogenic differentiation of tendon-derived stem cells, thus preventing tendon healing [25]. Several studies have also shown that the classical inflammatory pathway NF-κB signaling is activated during the pathogenesis of tendon senescence [24,26,27]. While inhibiting the NF-κB pathway reduces the release of pro-inflammatory mediators, tendinopathy progression can be delayed [27]. Therefore, targeting the NF-κB pathway to inhibit inflammation could be a feasible and effective method for treating tendinopathy.

Friedelin (FR) is a triterpenoid compound existing in many plants, which can be derived from Aristotelia chilensis leaves (Elaeocarpaceae), Cannabis roots, and Maytenus ilicifolia leaves [28]. Especially, FR is most abundant in the cork of trees [29]. FR has a wide range of biological activities, such as anti-inflammatory [30], analgesic, antioxidant [31], antitumor [32], and antibacterial effects [33]. Thus, it can be used to treat a variety of diseases, such as ulcerative colitis [28], gastric ulcer [34], and leukemia. Compared with non-steroidal anti-inflammatory drugs, the gastric protective function of FR has significant advantages [34]. Importantly, in the acute toxicity test in rats, FR showed good drug safety performance, and the maximum dosage was up to 80 mg/kg [34].

In this study, we found that FR ameliorated the structural disorder of the Achilles tendon, improved the biomechanical properties of the Achilles tendon, and attenuated inflammatory infiltration, thereby delaying the progression of tendinopathy in mice. Mechanistically, FR increases K48-linked ubiquitination of p65 by recruiting RNF182, promotes the binding of p62 and p65, and subsequently accelerates autophagic degradation of p65, thus inhibiting the NF-κB signaling pathway to reduce the tendon’s inflammatory response. Therefore, this study identifies that FR could be a potential drug for the prevention and treatment of tendinopathy.

## 2. Materials and Methods

### 2.1. Reagents and Antibodies

Reagents and antibodies in this study are shown in Table 1.

### 2.2. Animals and Treatment

This type of study belongs to basic experimental research. Eight-week-old C57BL/6 male mice were purchased from the Animal Center of Guangdong, Guangzhou, China. Care and use of all animals conformed to the guidelines set forth by the Chinese National Institutes of Health. Ethical approval for this study was obtained from the Medical Ethics Committee of the Medical College of Shantou University (No. SUMC2021-480). The experimental mice were randomly divided into four groups: a sham group, a collagenase-induced tendinopathy (CIT) group, a CIT model + FR group, and an FR group. The model of CIT was induced as previously described [35]. In short, 20 μL type I collagenase (1%) was injected around the right Achilles tendon of mice. One week after establishing the CIT model, the mice were treated with corresponding treatments. Specifically, the CIT + FR group was treated with a local injection of FR (40 μM, 20 μL) near the right Achilles tendon in CIT mice. Mice in the sham group and CIT group were injected with the same dose of saline. FR group mice were treated with a local injection of FR (40 μM, 20 μL) near the right Achilles tendon of normal mice. Four weeks after treatment, mice were euthanized and sampled, and the right Achilles tendons connected with the tibia and calcaneus were obtained for subsequent experimental study.

### 2.3. Biomechanical Assay 

We performed biomechanical testing as in a previous study [36]. Briefly, the collected Achilles tendon tissue (retaining the calcaneus and the lower 1/3 muscle of the triceps of the lower leg) was taken out for the biomechanical test. Then, the tensile test was carried out on the Instron 5943 dynamic and static test system of universal electronic materials (Instron Corporation, Canton, MA, USA). Both ends of the Achilles tendon were wrapped with saline gauze and then placed on the test apparatus fixture. After the operating parameters were entered, the biomechanical testing started.

### 2.4. Histological Assessments

The collected Achilles tendons were washed twice with PBS and then fixed with 4% paraformaldehyde (Sigma-Aldrich, St. Louis, MO, USA) at 4 °C for 24 h. Next, the samples were dehydrated, paraffin-embedded, and finally sliced with a thickness of 5 μm. The protocol of hematoxylin and eosin (HE) staining was carried out with reference to the previous research method. Briefly, Achilles tendon sections were dewaxed and hydrated, followed by hematoxylin staining for 5 min. After washing with PBS, eosin staining was performed for 3 min, and finally dried and sealed for storage. We referenced the established histological scoring system to analyze the changes in total histological scores on HE-stained slides after treatment [37]. The score of the intact group was defined as 20 points.

The staining instructions for the commercial Masson’s trichrome kit were followed for Masson staining. That is, Masson’s trichrome staining is used to visualize the original high-intensity collagen (red) and the newly synthesized low-intensity collagen (blue) [38].

For immunofluorescence staining, the Achilles tendon slices were first dewaxed and rehydrated. After washing with PBS three times, the sections were subjected to antigen retrieval with sodium citrate solution, followed by blocking with goat serum for 1 h. Then, primary antibodies were added and incubated overnight at 4 °C. On the second day, sections were incubated with the second antibody in the dark for 1 h and finally preserved by DAPI sealing. The scanning tissue microscope obtained different histological images (Olympus BX51, Tokyo, Japan). All histological images were obtained randomly at least 3 times.

### 2.5. Quantitative Reverse Transcription PCR (qRT-PCR) Assay

Total RNA was extracted from tissues or cells by TRIZOL. Next, we use reverse transcriptase to generate complementary cDNA. Real-time PCR was carried out using the ABI Q6 analyzer using the SYBR GreenER qRT-PCR SuperMix Universal and specific primers. Quantification of the gene expressions was assessed by fold changes normalized to the housekeeping gene GAPDH. The primers used in this study are listed in Table 2.

### 2.6. Cell Culture and Treatment

We extracted tenocytes according to previously researched methods [27]. Briefly, mice were first euthanized. Next, the bilateral Achilles tendons of mice were isolated, and the adipose tissue and muscle tissue around the Achilles tendon were carefully removed. After washing the Achilles tendon with PBS three times, the Achilles tendon was cut into pieces with ophthalmic scissors and placed in a DMEM medium. Subsequently, type I collagenase was added to the chopped Achilles tendon for digestion for 3 h. The digested tissue was passed through a 100 μm cell filter and then centrifuged at 400× *g* for 5 min. The supernatant was discarded, and the cells were suspended in a complete medium (DMEM with 10% fetal bovine serum, 1% penicillin/streptomycin, and 1% amphotericin B) and cultured in an incubator at 37 °C, 5% carbon dioxide and 95% humidity. The medium was changed every three days, and the cells were passaged after trypsinization. When the cells were cultured to the third passage, they were used for subsequent experimental research.

For the stimulation of tenocytes, the cells were plated in 6-well plates at a density of 2 × 10^6^ cells per well, and then 1.5 mL of complete medium was added. After 24 h of culture, the cells were pretreated with different concentrations of FR for 3 h. Subsequently, the cells were treated with IL-1β (10 ng/mL; R&D Systems, Minneapolis, MN, USA) for another 24 h. Finally, the cell supernatant was collected, and cellular RNA and protein were extracted for subsequent experiments.

### 2.7. Cell Viability Assay

We used a Cell Counting Kit-8 (CCK-8) to evaluate cell proliferation and viability. In short, tenocytes were seeded in 96-well plates at a density of 1 × 10^3^/well. After the cells adhered, they were treated with different concentrations of FR for 24 h. 10 μL The CCK-8 working solution was added to each well and cultured in the cell incubator for 4 h. Finally, the absorbance value was measured in a microplate reader with a wavelength of 450 nm.

### 2.8. Plasmids and Transfection

Plasmids were cloned into the pcDNA3.1 vector for transient expression. HEK293T transfection was performed using Lipofectamine 2000 according to procedures recommended by the manufacturer. Chemically synthesized 21-nucleotide siRNA duplexes were obtained from TranSheepBio and transfected using Lipofectamine RNAiMAX according to the manufacturer’s instructions. The sequences of target siRNAs are shown in Table 3. 

### 2.9. Protein Degradation Inhibition Assays

MG132 (10 μM) was used to inhibit proteasome-mediated protein degradation. Both 3-MA (10 mM) and CQ (50 μM) were used to inhibit autolysosome- or lysosome-mediated protein degradation.

### 2.10. ELISA

Cell supernatants and serum were detected by the mouse IL-1β, IL-6, and TNF-α ELISA kit (#E-EL-M0037c, #E-EL-M0044c, #E-EL-M1084c; Elabscience Biotechnology Co., Ltd., Wuhan, China). Absorbance was detected at 450 nm by the Multiskan FC (Thermo Fisher, Waltham, MA, USA).

### 2.11. Western Blotting

Proteins from cells and tissues were extracted with cell lysates (Tris-HCI, pH7.5, 1 M; EDTA 0.5 M; 10% SDS; NP-40; sodium deoxycholate; CHAPS Triton X-100). The lysed protein liquid was then placed in the EP tube and centrifuged at 13,000 rpm at 4 °C for 5 min. Next, we took the protein supernatant, added the loading buffer, and boiled it in a metal bath for 15 min to denaturate the protein. After the protein concentration was determined, it was packed and stored at −20 °C. Then the electrophoretic gel was prepared, and the protein samples were added to the gel for electrophoresis. The gel was taken out and placed in PVDF membrane for transfer reaction (100 V, 90 min). The PVDF membrane was placed in 5% skim milk and sealed for 1 h. Then, the primary antibodies were added and incubated overnight. The next day, the secondary antibodies were added and incubated for 1 h. Finally, signals were revealed using an enhanced chemiluminescence kit.

### 2.12. Statistical Analysis

The data were graphed using GraphPad Prism software version 8.0 (GraphPad Software Inc., La Jolla, CA, USA). We used a one-way analysis of variance followed by Student’s t-test to determine statistical differences between treatment groups. Error bars represent the standard error of the mean in the cell experiment and the standard deviation in the animal experiment. Differences between the two groups were considered significant when the *p*-value was less than 0.05.

## 3. Results

### 3.1. FR Alleviates the Progression of Tendinopathy in Mice

**Figure 1:**
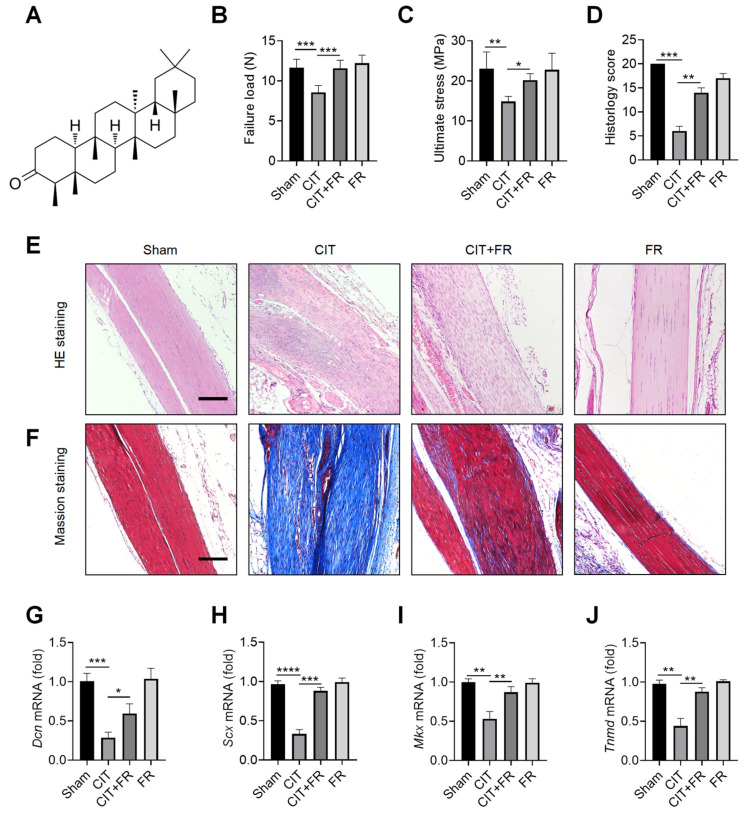
Progression of tendinopathy is alleviated by FR in mice. (**A**) The chemical structural formula of FR. Four weeks after treatment in mice, the Achilles tendons were collected for experimental detection. Biomechanical detection of the effect of FR on Achilles tendon healing: failure load (**B**), ultimate stress (**C**). (**D**) Histological score of HE staining. (**E**) HE staining was used to detect the effect of FR on the histological healing of the Achilles tendon in mice. (**F**) Masson’s staining was used to detect the effect of FR on the formation of collagen fibers in the process of tendinopathy in mice. (**G**–**J**) The effect of FR on the expression of tendon-forming factors (*Dcn*, *Scx*, *Mkx*, and *Tnmd*) was detected by qRT-PCR. Scale bar: 100 µm. Data are expressed as the means ± SD from three independent experiments. * *p* < 0.05, ** *p* < 0.01, *** *p* < 0.001, **** *p* < 0.0001.

To explore the effect of FR on the progression of tendinopathy, we first constructed a mouse model of CIT. The chemical structural formula of FR is shown in Figure 1A. In the CIT group, the biomechanical indexes decreased, the histological structure was disordered, and the transcription level of tenogenic factors decreased, indicating that the CIT mouse model was successful. Compared with the CIT group, FR significantly increased the failure load (Figure 1B) and ultimate stress (Figure 1C) in the CIT + FR group. However, the biomechanical indexes of tendons in the FR group were not affected by FR alone (Figure 1B,C). In addition, the HE staining indicated that FR effectively alleviated the structural disorder of tendinopathy, reduced inflammatory cell infiltration, restored the normal arrangement of collagen fibers, and reduced neovascularization (Figure 1E). Similarly, histological scoring results showed a consistent trend (Figure 1D). In addition, the results of Masson staining showed that a large number of low-strength collagen fibers (blue part) were produced in the CIT group, while more high-strength collagen fibers (red part) were produced in the CIT + FR group (Figure 1F). Moreover, FR alone did not affect the formation of collagen fibers, which indicates that FR functions during the progression of tendinopathy. To further clarify the effect of FR on tenogenesis in tendinopathy, we detected the transcriptional levels of tendon forming factors (*Dcn*, *Scx*, *Mkx* and *Tnmd*) and found that FR effectively reversed the mRNA expression of *Dcn*, *Scx*, *Mkx* and *Tnmd* in mice with tendinopathy (Figure 1G–J). Therefore, these data demonstrate that FR effectively alleviates the progress of tendinopathy in mice.

### 3.2. FR Attenuates the Infiltration of Inflammatory Factors during Tendinopathy

**Figure 2:**
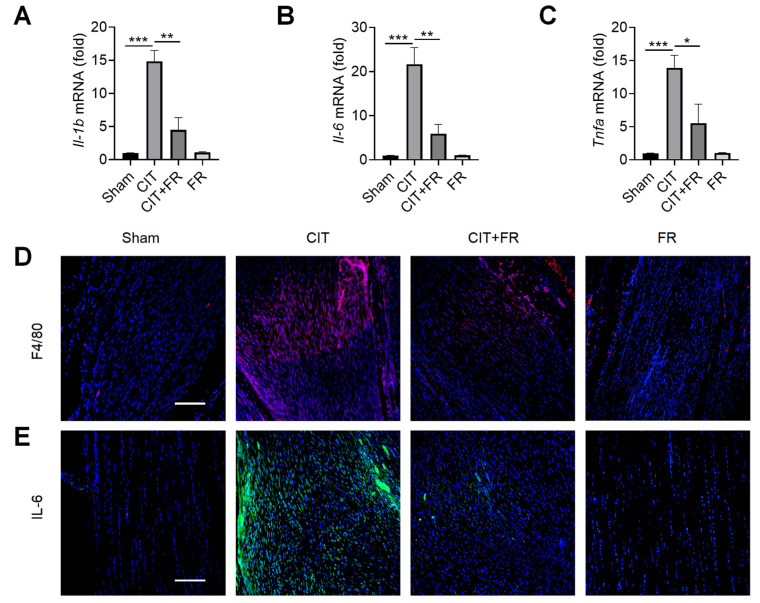
FR alleviates the inflammatory response of tendinopathy in mice. (**A**–**C**) Four weeks after treatment in mice, the effect of FR on the expression of inflammatory cytokine (*Il-1b*, *Il-6*, and *Tnfa*) mRNA was detected by qRT-PCR. The expressions of macrophage marker F4/80 (**D**) and inflammatory factor IL-6 (**E**) were detected by immunofluorescence. Scale bar: 100 µm. Data are expressed as the means ± SD from three independent experiments. * *p* < 0.05, ** *p* < 0.01, *** *p* < 0.001.

Inflammation is known to play an important role in the progression of tendinopathy [39]. Next, we investigated the effect of FR on the inflammatory response of tendinopathy in mice. qRT-PCR results showed that the expression of inflammatory cytokines *Il-1b*, *Il-6* and *Tnfa* significantly increased during tendinopathy, and this enhancement was reversed by FR treatment in the CIT group (Figure 2A–C). Macrophages, the key immune cells that mediate inflammation, play an important pathogenic role in the progression of tendinopathy [40]. Then, we explored whether FR could regulate the infiltration of macrophages in the process of tendinopathy. As shown in Figure 2D, a large number of F4/80^+^ macrophages were infiltrated in the CIT group, while the number of F4/80^+^ macrophages was dramatically reduced in the CIT + FR group. Consistent with this, the immunofluorescence result showed that the protein expression of IL-6 in the CIT + FR group was significantly lower than in the CIT group (Figure 2E). Moreover, FR alone did not affect the expression of F4/80^+^ macrophages and IL-6 in normal mice. These data suggest that FR attenuates the infiltration of inflammatory cytokines and cells during tendinopathy in mice, thereby attenuating the progression of tendinopathy.

### 3.3. FR Targets p65 to Regulate NF-κB Signaling to Inhibit the Inflammatory Response in Tenocytes

**Figure 3:**
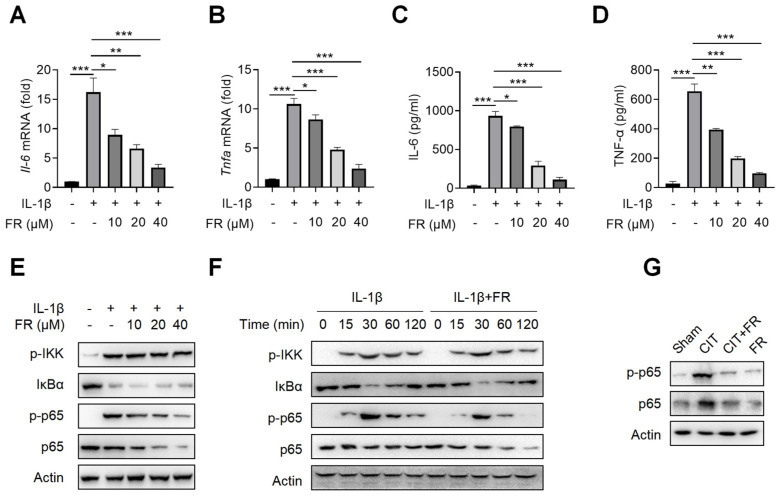
FR targets p65 to inhibit NF-κB signaling in vitro and *in vivo*. Tenocytes were stimulated with IL-1β (10 ng/mL) and FR (0–40 μM) for 24 h. The cell supernatant, total cell RNA, and total protein were collected for experimental detection. qRT-PCR was used to detect the effect of FR on the mRNA expression of *Il-6* (**A**) and *Tnfa* (**B**) mediated by IL-1β. ELISA was used to detect the effect of FR on the supernatant protein expression of IL-6 (**C**) and TNF-α (**D**) induced by IL-1β. (**E**) The expression of NF-κB pathway-related proteins (p-IKK, IκBα, p-p65, and p65) treated with different concentrations of FR was detected by WB. (**F**) Tenocytes were treated with IL-1β (10 ng/mL) and FR (40 μM) for 0–120 min. The expression of NF-κB pathway-related proteins (p-IKK, IκBα, p-p65, p65) was detected by WB. (**G**). Four weeks after treatment, the protein expression of p-p65 and p65 in Achilles tendons was detected by WB. The data are representative of three independent experiments. Error bars show the means ± SD. * *p* < 0.05, ** *p* < 0.01, *** *p* < 0.001.

To investigate the mechanism of FR in inhibiting inflammation, we then treated the tenocytes with FR under the condition of inflammatory stimulation. Firstly, the CCK-8 result suggests that the concentrations of 0–40 μM of FR have no effect on the proliferation of tenocytes (Figure S1A), which indicates that this is the safe concentration range for the subsequent experiments. It is reported that IL-1β is highly expressed in tendinopathy, which is a key inflammatory mediator to mediate the inflammatory reaction and accelerate tendinopathy [39,41]. We then explored the effect of FR on the IL-1β-mediated inflammatory response of tenocytes and found that FR reduces the mRNA expression of *Il-6* and *Tnfa* induced by IL-1β (Figure 3A,B). Meanwhile, FR also inhibited the protein expression of IL-6 and TNF-α proteins mediated by IL-1β (Figure 3C,D). These data indicated that FR effectively inhibits the IL-1β-mediated release of inflammatory factors in tenocytes. In addition, we also examined the effect of FR on the inflammatory responses mediated by other inflammatory factors. Both LPS- and TNF-α-mediated inflammation can be effectively alleviated by FR (Figure S1B–E). Therefore, FR can effectively inhibit the inflammatory response of tenocytes induced by various inflammatory mediators.

It is known that NF-κB signaling is not only one of the most classical pathways to mediate the inflammatory response but also the key pathway to aggravating the deterioration of tendinopathy [24]. We further investigated whether the anti-inflammatory effect of FR is achieved by targeting the NF-κB pathway. As shown in Figure 3E, FR inhibited the expression and phosphorylation of p65 in a concentration-dependent manner but did not affect the expression of p-IKK and IkBα. Similarly, FR also inhibited the expression of p65 and p-p65 without affecting the expression of p-IKK and IkBα after different times of stimulation (Figure 3F). Consistent results were also obtained in the condition of LPS or TNF-α mediated inflammation (Figure S1F–H). Furthermore, it was confirmed in vivo that FR inhibited the expression of p65 and p-p65 (Figure 3G). These data suggest that FR targets the NF-κB pathway and plays an anti-inflammatory role in tenocytes at the molecular level of p65.

### 3.4. FR Promotes the Degradation of p65 through the Autophagy-Lysosome Pathway

**Figure 4:**
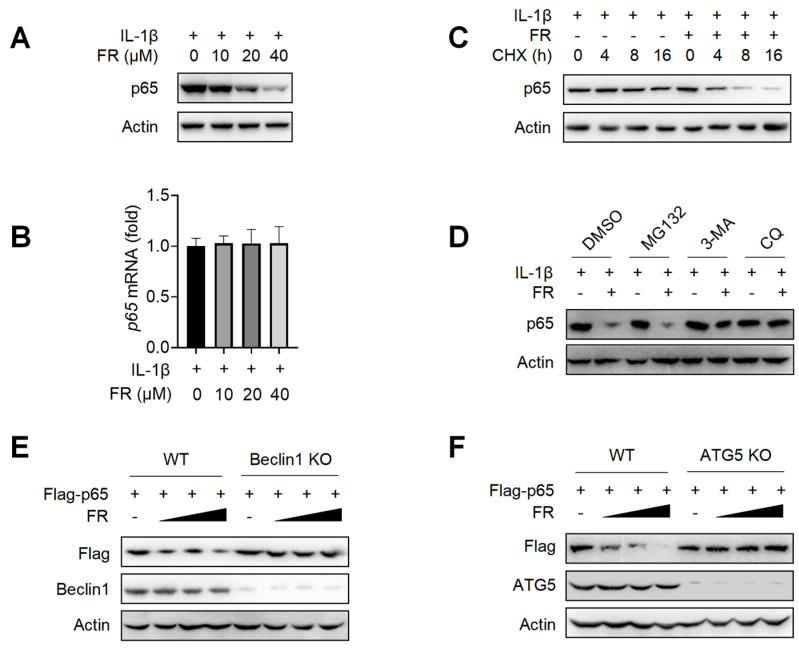
FR degrades p65 through an autophagy-lysosome pathway. Tenocytes were treated with IL-1β (10 ng/mL) and FR (40 μM) for 24 h. (**A**) The expression of p65 protein was detected by WB. (**B**) The mRNA expression of *p65* was detected by qRT-PCR. (**C**) Tenocytes were treated with CHX (50 μg/mL), IL-1β (10 ng/mL) and FR (40 μM) for 0–16 h. The protein expression of p65 was detected by WB. (**D**). Tenocytes were pretreated with MG132 (10 μM), or 3-MA (5 mM), or CQ (10 μM) for 6 h, followed by the addition of IL-1β (10 ng/mL) and FR (40 μM) for 24 h, and then the p65 protein was detected by WB. (**E**) WT and Beclin1-knockout HEK293T cells were transfected with Flag-p65 plasmid for 24 h, followed by 8 h treatment with FR (0–40 μM). Finally, the protein expression of Flag-p65 and Beclin1 were detected by WB. (**F**) WT and ATG5-knockout HEK293T cells were transfected with Flag-p65 plasmid for 24 h, followed by 8 h treatment with FR (0–40 μM). Then, the protein expression of Flag-p65 and ATG5 were detected by WB. Data are expressed as the means ± SD from three independent experiments.

Next, we further explore the mechanism of FR in inhibiting the expression of p65. We found that FR inhibited the expression of p65 protein (Figure 4A) but did not affect the transcription level of p65 (Figure 4B). These data suggest that FR may inhibit the expression of p65 by regulating the degradation of p65. To further confirm that FR promotes the degradation of p65, we investigated the effect of FR on the degradation of p65 under the condition of protein synthesis inhibitor cycloheximide (CHX). As shown in Figure 4C, FR accelerated the degradation of p65 in the presence of CHX, suggesting that FR indeed plays a role in promoting the degradation of p65.

Since there are at least three protein degradation systems (the proteasome, lysosome, and autolysosome pathways) [42], we next determined for which pathway FR induces the degradation of p65. The degradation of p65 by FR was reversed when the autophagy inhibitor 3-MA and the lysosomal inhibitor CQ were used, but not by the proteasome inhibitor MG132 (Figure 4D). To further confirm that FR degrades p65 protein through the autophagy-dependent pathway, we used Beclin1 deficiency or ATG5 deficiency HEK293T cells for verification. In WT HEK293T cells, FR significantly promoted the degradation of exogenous p65 protein. However, when Beclin1 or ATG5 was knocked out in HEK293T, the degradation of p65 by FR disappeared (Figure 4E,F). These results demonstrated that FR promotes the degradation of p65 through an autophagy-lysosome pathway.

### 3.5. FR Mediates Selective Autophagic Degradation of p65 via p62-Dependent Pathway

**Figure 5:**
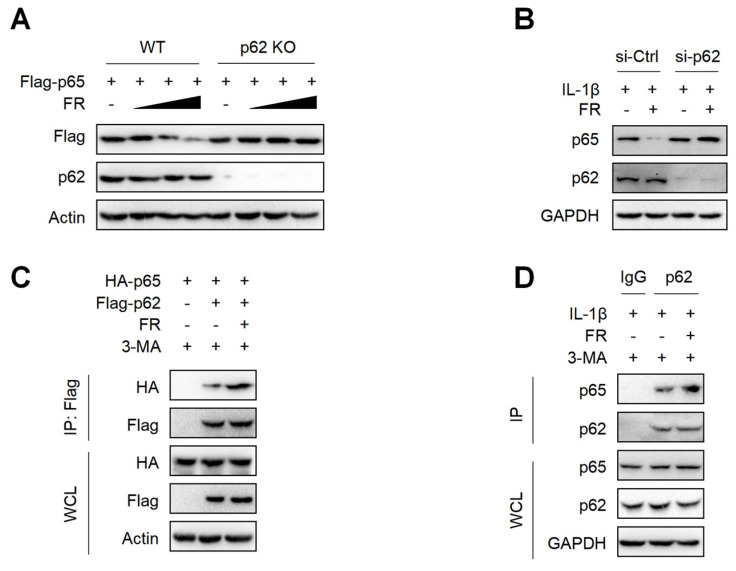
FR promotes the degradation of p65 via p62-mediated selective autophagy. (**A**) The Flag-p65 plasmid was transfected into WT and p62 knockout HEK293T cells for 24 h, and FR (0–40 μM) was added for another 8 h. Finally, WB was used to detect the protein expression of Flag-p65 and p62. (**B**) Tenocytes were transfected with p62 siRNA for 24 h and then treated with IL-1β (10 ng/mL) and FR (40 μM) for 24 h. Finally, the protein expression of p65 and p62 was detected by WB. (**C**) HEK293T cells were transfected with HA-p65 and Flag-p62 plasmids for 24 h, then treated with 3-MA (5 mM) for 6 h, and then treated with FR (40 μM) for another 8 h. Finally, cell proteins were collected for immunoprecipitation (IP). The expressions of HA-p65 and Flag-p62 proteins in IP samples and whole-cell lysates (WCL) were detected by WB. (**D**) Tenocytes were pretreated with 3-MA (5 mM) for 6 h, then stimulated with IL-1β (10 ng/mL) and FR (40 μM) for 24 h, and the cellular proteins were collected for IP. WB was used to detect the protein expression of p65 and p62 in IP samples and WCL. Data are expressed as the means ± SD from three independent experiments.

p62 is a selective autophagy adaptor protein, which plays an important role in mediating the autophagic degradation of proteins [42,43]. Next, we explored whether FR mediates the selective autophagic degradation of p65 through p62. The results showed that FR could not degrade p65 in p62-knockout HEK293T cells (Figure 5A). Moreover, silencing p62 in tenocytes could also reverse the degradation of p65 by FR (Figure 5B). Therefore, FR promoted the degradation of p65 via p62-mediated selective autophagy. Subsequently, we further explored whether FR affected the degradation of p65 by influencing the interaction between p62 and p65. In the exogenous IP experiment, FR promoted the interaction between p62 and p65 (Figure 5C). In addition, FR also enhanced the association of endogenous p62 and p65 (Figure 5D). These results suggest that FR promotes the selective autophagic degradation of p65 by increasing the association of p62 and p65.

### 3.6. FR Increases the K48-Linked Ubiquitination of p65 to Promote Its Autophagic Degradation

**Figure 6:**
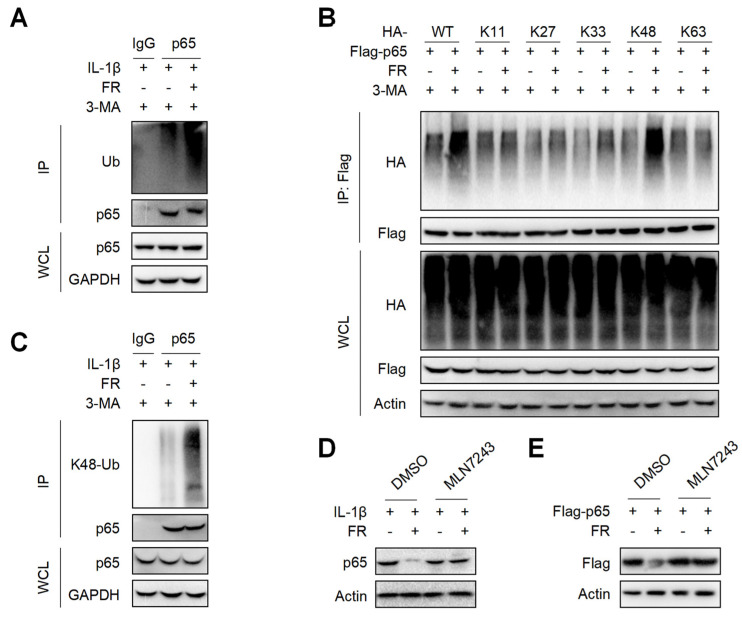
FR promotes the K48-linked ubiquitination of p65 in vitro. (**A**) Tendon cells were pretreated with 3-MA (5 mM) for 6 h, then stimulated with IL-1β (10 ng/mL) and FR (40 μM) for 24 h, and cell proteins were collected for IP. WB was used to detect the protein expression of p65 and Ub. (**B**) Flag-p65 and HA-labeled ubiquitinated plasmids (WT, K11, K27, K33, K48, K63) were transfected into HEK293T cells for 24 h, then treated with 3-MA (5 mM) for 6 h, and then treated with FR (40 μM) for 8 h. Finally, the proteins were collected for IP. WB was used to detect the protein expression with HA tag and Flag tag in IP samples and WCL. (**C**) Tenocytes were pretreated with 3-MA (5 mM) for 6 h, then stimulated with IL-1β (10 ng/mL) and FR (40 μM) for 24 h, and cell proteins were collected for IP. WB was used to detect the protein expression of p65 and K48 Ub in IP samples and WCL. (**D**) Tenocytes were pretreated with ubiquitination inhibitor MLN7243 (5 μM) for 6 h, then treated with IL-1β (10 ng/mL) and FR (40 μM) for 24 h, and the expression of p65 protein was detected by WB. (**E**) Flag-p65 plasmid was transfected into HEK293T cells for 24 h, then MLN7243 (5 μM) was added for 6 h, and FR (40 μM) was added for 8 h. WB was used to detect the protein expression of Flag-p65. Data are expressed as the means ± SD from three independent experiments.

It is reported that p62 directs ubiquitinated proteins to autophagolysosomes for selective degradation mainly through its C-terminal ubiquitin-associated domain [42,43]. Next, we investigated whether FR could affect the ubiquitination of p65 and found that FR remarkably increased the poly-ubiquitination of endogenous p65 (Figure 6A). Subsequently, we found that FR specifically increased K48-linked (K48-only ubiquitin mutant) poly-ubiquitination of p65, but not the ubiquitination of p65 with other ubiquitin linkages in an overexpression system (Figure 6B). Likewise, FR also promoted the K48-linked ubiquitination of endogenous p65 (Figure 6C). In addition, the degradation of p65 by FR was reversed in the presence of ubiquitin inhibitor MLN7243 (Figure 6D,E). These data suggest that FR accelerates p62-mediated selective degradation by promoting the K48-linked ubiquitination of p65.

### 3.7. FR Mediates K48-Linked Ubiquitination of p65 via E3 Ubiquitination Enzyme RNF182

**Figure 7:**
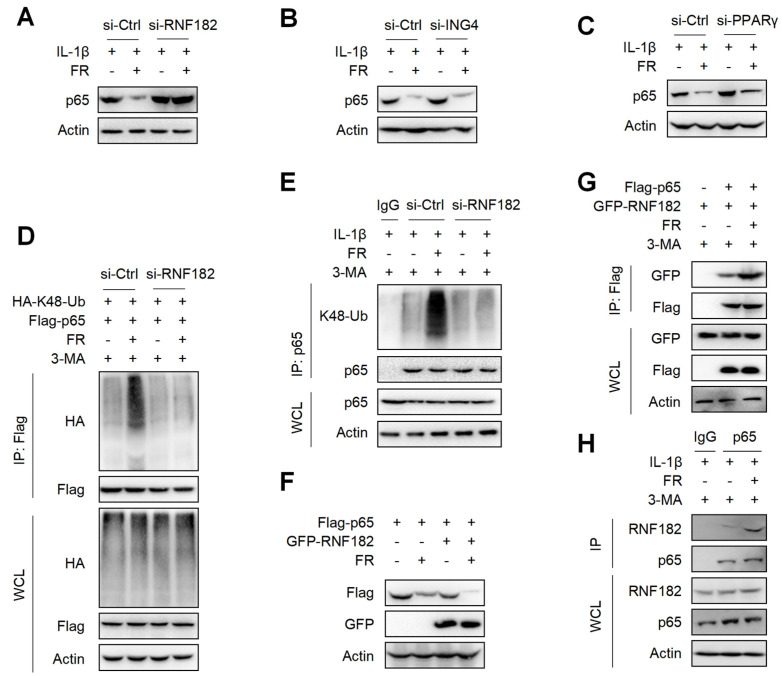
FR promotes the K48-linked ubiquitination of p65 by recruiting the E3 ubiquitin enzyme RNF182. Tenocytes were silenced with RNF182 siRNA (**A**), ING4 siRNA (**B**), or PPARγ siRNA (**C**) for 24 h and then treated with IL-1β (10 ng/mL) and FR (40 μM) for 24 h. Finally, the protein expression of p65 was detected by WB. (**D**) HEK293T cells were treated with RNF182 siRNA for 24 h, then transfected with HA-K48-Ub and Flag-p65 plasmids for 12 h, and next stimulated with 3-MA (5 mM) for 6 h, and finally treated with FR (40 μM) for 8 h. Cell lysates were collected for IP and WB detection. (**E**) Tenocytes were transfected with RNF182 siRNA for 24 h, then treated with 3-MA (5 mM) for 6 h, and finally treated with IL-1β (10 ng/mL) and FR (40 μM) for 24 h. Cell proteins were collected for IP. The expressions of p65 and K48-Ub in IP and WCL samples were detected by WB. (**F**) Flag-p65 and GFP-RNF182 plasmids were transfected into HEK293T cells for 24 h and then treated with FR (40 μM) for 8 h. Finally, WB was used to detect the protein of the Flag tag and GFP tag. (**G**) HEK293T cells were transfected with Flag-p65 and GFP-RNF182 plasmids for 24 h, then 3-MA (5 mM) was added for 6 h and then treated with FR (40 μM) for another 8 h. Finally, cell proteins were collected for IP, and proteins with Flag and GFP labels were detected by WB. (**H**) Tenocytes were pretreated with 3-MA (5 mM) for 6 h, followed by the addition of IL-1β (10 ng/mL) and FR (40 μM) for 24 h. Cell proteins were collected for IP. WB detected the protein expressions of p65 and RNF182 in IP and WCL samples. Data are expressed as the means ± SD from three independent experiments.

It is reported that E3 ubiquitinase is a key enzyme mediating protein ubiquitination [44]. Although FR promotes the ubiquitination of p65, it is not clear which E3 ubiquitin enzyme plays a key role in the FR-induced ubiquitination of p65. It is reported that RNF182, ING4, and PPARγ are E3 ubiquitination enzymes that mediate the K48-linked ubiquitination of p65 [45,46,47]. Next, we determined which E3 ubiquitin enzyme plays its role in the FR-mediated ubiquitination of p65. We silenced the expression of RNF182, ING4, and PPARγ in tenocytes by the siRNAs (Figure S2A–C) and found that silencing RNF182 reversed the degradation of p65 by FR (Figure 7A), whereas silencing ING4 and PPARγ did not reverse the degradation of p65 (Figure 7B,C), which indicates that FR mediates the degradation of p65 through the RNF182-dependent pathway. Next, we examined whether FR mediated the K48-linked ubiquitination of p65 through RNF182 and found that FR failed to promote the K48-linked ubiquitination of p65 when RNF182 was silenced in the overexpression system (Figure 7D). Meanwhile, similar results were obtained in the endogenous IP experiment (Figure 7E). In addition, in the case of overexpression of RNF182, FR could further promote the degradation of p65 (Figure 7F). These data suggest that FR mediates the K48-linked ubiquitination of p65 by recruiting RNF182, promoting autophagic degradation. Furthermore, we also found that FR effectively increased the interaction between RNF182 and p65 in the overexpression and endogenous systems (Figure 7G,H). Therefore, these data suggest that FR promotes the K48-linked ubiquitination of p65 by promoting the association of RNF182 and p65, thus accelerating the autophagic degradation of p65.

## 4. Discussion

**Figure 8:**
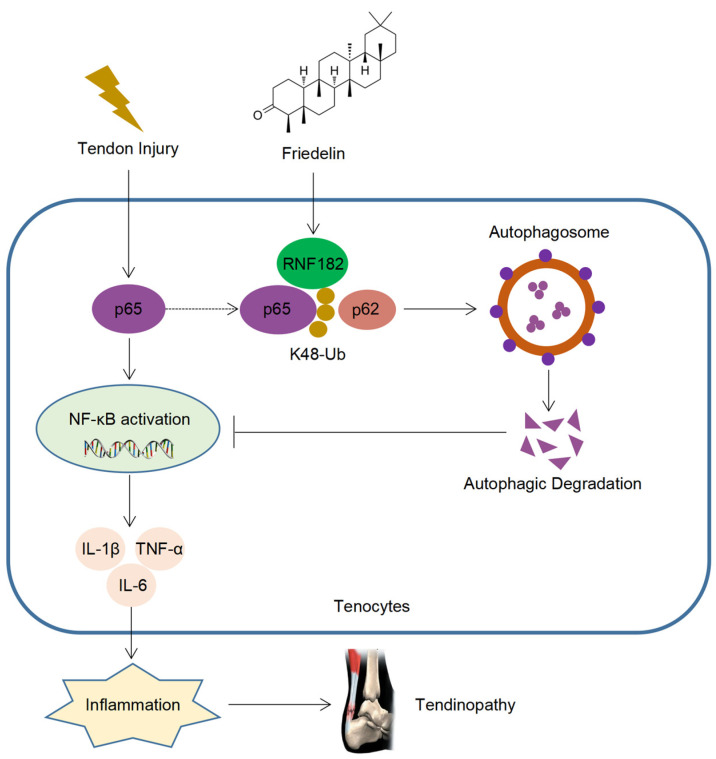
Schematic diagram of the action of FR. FR promotes the K48-linked ubiquitination of p65 through recruiting E3 ubiquitinase RNF182 and then enhances p62-mediated selective autophagic degradation of p65, thereby inhibiting NF-κB signaling and alleviating the progression of tendinopathy.

Tendinopathy is a degenerative disease that occurs after tendon injury, which often causes local pain and limited mobility and leads to disability in severe cases. With the development of aging, the incidence of tendinopathy is increasing year by year [3,4,5]. However, there is currently no cure for tendinopathy, which brings significant challenges to the prevention and treatment of tendinopathy. FR is a triterpenoid compound with a wide range of sources, high safety, and a variety of medicinal values [28,48,49]. Although FR plays a certain role in treating inflammatory diseases, there is a lack of in-depth research on the target and mechanisms of its disease resistance. Here, we found that FR alleviated the degeneration of tendinopathy, reduced inflammatory infiltration, and improved the biomechanical strength, which plays an important role in the prevention and treatment of tendinopathy. Mechanically, FR inhibited the NF-κB pathway by promoting the autophagic degradation of p65 and decreasing the inflammatory response, thereby alleviating the progression of tendinopathy. Furthermore, FR increased the K48-linked ubiquitination of p65 by recruiting the E3 ubiquitinase RNF182, promoting the association of p65 and cargo protein p62 and thus mediating the selective autophagic degradation of p65 (Figure 8).

After tendon trauma, a large number of immune cells can be recruited to the local area, in which macrophages release a variety of inflammatory factors to mediate the chronic inflammation of tendinopathy [13,40,50]. It is reported that a small number of inflammatory factors could mediate the regeneration of tendon tissue, but long-term chronic inflammation will lead to tendon tissue degeneration [13,40,50]. In addition, chronic inflammation can further activate relevant immune cells, produce various pathogenic factors, and hinder the repair and healing of tendon tissue [17,23,51]. In the long run, normal tendon tissue is replaced by hyperplastic tissue and even calcification [52,53]. Therefore, targeted elimination of inflammation is a good strategy for preventing and treating tendinopathy. However, drugs that target inflammation and their mechanisms remain unknown, limiting the development of tendinopathy treatments. Here, our results show that FR improves the biomechanical strength of the tendon and promotes the generation of high-strength muscle fibers, which are important for preventing tendon rupture. Meanwhile, FR reduces tendon fibrosis, restores the orderly arrangement of collagen fibers, and decreases angiogenesis in tendinopathy. FR effectively inhibits the expression of inflammatory factors and reduces the infiltration of F4/80^+^ macrophages. More importantly, FR can also promote the expression of tendon-forming factors (*Scx*, *Tnmd* and *Mkx*). In addition, the use of FR alone does not cause pathological changes in the normal tendon. These findings show that FR is a safe and effective candidate for treating tendon diseases.

Several studies have shown that the NF-κB pathway is a classical pathway that mediates inflammatory cascade [27,54,55]. During the progression of tendinopathy, the NF-κB pathway is significantly activated, leading to an inflammatory storm and accelerating the progression of tendinopathy [27,54,55]. It is reported that the progression of tendinopathy is weakened in IKKβ-deficient mice, indicating targeted inhibition of the NF-κB pathway is a good strategy for the prevention and treatment of tendinopathy [27]. Our study found that FR can inhibit the NF-κB pathway, thus inhibiting the release of inflammatory factors. Unlike other anti-inflammatory drugs, FR acts on p65 molecules downstream of the NF-κB pathway without affecting the upstream pathway, which shows good selectivity and targeting. To clarify the pharmacological mechanism of the anti-inflammatory effect of FR, we conducted experiments in tenocytes and HEK293T cells. The results showed that FR promoted the autophagy-dependent degradation of p65 but did not affect the transcription of p65. This indicates that FR inhibits the NF-κB pathway by regulating the post-translational modification of p65. Further results showed that FR mediated the autophagic degradation of p65 through autophagy cargo p62. Moreover, FR promotes the interaction of p65 and p62. Therefore, these results demonstrate that FR promotes autophagic degradation by increasing the combination of p62 and p65.

Ubiquitination is a form of post-translational modification of proteins by coupling ubiquitin proteins to substrate proteins [56,57,58]. Ubiquitin is a protein containing 76 amino acids that bind to the lysine residue of the substrate protein by an isopeptide bond [56,57,58]. Ubiquitin contains seven lysine residues (K6, K11, K27, K29, K33, K48 and K63) and one methionine [56,57,58]. Since p62 needs to be combined with ubiquitinated substrates [42], we further explored how FR regulates the ubiquitination of p65. Our results showed that FR could promote the K48-linked ubiquitination of p65. In addition, after blocking ubiquitination, p65 could not be degraded by FR, which indicated that the degradation of p65 by FR was dependent on ubiquitination. Therefore, targeted regulation of ubiquitination is also one of the effective strategies for the treatment of tendinopathy. Ubiquitination occurs through the sequential activities of three enzymes, namely ubiquitin enzyme (E1), ubiquitin-binding enzyme (E2), and ubiquitin ligase (E3). Several E3 ubiquitin enzymes have been reported to mediate the K48-linked ubiquitination of p65 [45,46,47]. Here, we confirmed that FR mediates the K48-linked ubiquitin of p65 through the E3 ubiquitin enzyme RNF182 but is independent of ING4 and PPARγ. Moreover, the interaction between RNF182 and p65 can be enhanced by FR. Therefore, the targeted degradation of p65 by FR is closely related to p62 and RNF182, which provides a theoretical basis for applying FR in the treatment of tendinopathy and other inflammatory diseases.

Although the present study demonstrates that FR has a good alleviating effect on tendinopathy, some limitations still need to be further studied. Due to ethical issues, this study only used mouse tenocytes for experiments. However, further study is needed to determine whether FR has the same effect on human tenocytes. Additionally, although we have proven that FR shows a good anti-inflammatory effect on tendinopathy, whether it has a similar effect on other inflammatory bone diseases remains to be explored.

## 5. Conclusions

In conclusion, FR reduces the inflammatory response of tenocytes, improves the biological properties of the tendon, and promotes tendon healing by targeting the degradation of p65, thus effectively alleviating the progress of tendinopathy. To our knowledge, this study describes for the first time that FR alleviates tendinopathy by regulating ubiquitin-autophagy degradation. Therefore, FR is a promising new drug for treating tendinopathy and inflammation-related diseases. Of course, further clinical trials are needed to prove its safety and efficacy in human tendinopathy.

## Figures and Tables

**Table 1:** The resource of Reagents and Antibodies.

REAGENT or RESOURCE	SOURCE	IDENTIFIER
**Antibodies**		
Anti-F4/80	ABclonal	A18637
Anti-IL-6	Cell Signaling Technology	#12912
Anti-p-IKK	Cell Signaling Technology	#2697
Anti-IκBa	Cell Signaling Technology	#4814
Anti-p-p65	Cell Signaling Technology	#3039
Anti-p65	Santa Cruz Biotechnology	sc-8008
Anti-Actin	ABclonal	AC026
Anti-Flag agarose gels	Sigma-Aldrich	A2220
Horseradish peroxidase (HRP)-anti-Flag (M2)	Sigma-Aldrich	A8592
Anti-HA	Roche Applied Science	12013819001
Anti-Beclin1	Cell Signaling Technology	#3738
Anti-ATG5	Cell Signaling Technology	#12994
Anti-p62	Santa Cruz Biotechnology	sc-48402
Anti-Ub	Santa Cruz Biotechnology	sc-8017
Anti-K48 Ub	ABclonal	A3606
Anti-RNF182	Novus Biologicals	NBP1-82707
Anti-ING4	ABclonal	A5833
Anti-PPARγ	ABclonal	A11183
Goat anti-rabbit IgG (H&L)	Beijing Ray Antibody Biotech	RM3002
Goat anti-mouse IgG (H&L)	Beijing Ray Antibody Biotech	RM3001
Goat anti-rabbit IgG (H&L) Alexa Fluor 488	Immunoway	RS23220
Goat anti-rabbit IgG (H&L) Alexa Fluor 594	Immunoway	RS23420
Anti-IL-1β (ELISA)	Elabscience Biotechnology	#E-EL-M0037c
Anti-IL-6 (ELISA)	Elabscience Biotechnology	#E-EL-M0044c
Anti-TNF-α (ELISA)	Elabscience Biotechnology	#E-EL-M1084c
**Reagents**		
Friedelin	MedChemExpress	HY-N4110
LPS	Sigma-Aldrich	L2630
Recombinant IL-1β	Abcam	ab9723
Recombinant TNFα	Biovision	1051-1000
MG-132	Sigma-Aldrich	C-2211
Chloroquine	Glpbio	1954/5/7
Cycloheximide	Sigma-Aldrich	C7698
3-methyladenine	Sigma-Aldrich	5142-23-4
MLN7243	Sigma-Aldrich	HY-100487
DMSO	Sigma-Aldrich	D2650
Paraformaldehyde	Sigma-Aldrich	P6148
Lipofectamine 2000	ThermoFisher	11668019
Protein G beads	GenScript	L00209
Type I collagenase	Wako	031-17601
Hematoxylin and eosin solution	Beyotime	C0105S
Masson's trichrome kit	Solarbio	G1340
TRIZOL	Beyotime	R0016
Fetal bovine serum	Sigma-Aldrich	12103C
Penicillin/streptomycin	Gibco	15140122
Amphotericin B	Gibco	15290026
Trypsinization	Gibco	R001100
Cell Counting Kit-8	Beyotime	C0037
Lipofectamine 2000	Invitrogen	11668030
Lipofectamine RNAiMAX	Invitrogen	13778150
Enhanced chemiluminescence kit	Cell Signaling Technology	#12630

**Table 2:** Primers for real-time RT-PCR used in this study.

Gene	Sequences	Species
*Dcn*	Forward: AGACTCACAGCCGAGTAGGA Reverse: ACATTCGCATCTCAGACACC	Mouse
*Scx*	Forward: CCTTCTGCCTCAGCAACCAG Reverse: GGTCCAAAGTGGGGCTCTCCGTGACT	Mouse
*Mkx*	Forward: CCCCGGACATCGGATCTACTA Reverse: CTCTTAGGATGAGGATTTAGGTA	Mouse
*Tnmd*	Forward: GGGTGGTCCCGCAAGTGAAGGTG Reverse: GCCTCGACGACAGTAAATACAACAGT	Mouse
*Il-1b*	Forward: GCAACTGTTCCTGAACTCAACT Reverse: GTGCTCATGTCCTCATCCTG	Mouse
*Il-6*	Forward: CTCTGGGAAATCGTGGAAAT Reverse: CCAGTTTGGTAGCATCCATC	Mouse
*Tnfa*	Forward: GACGTGGAACTGGCAGAAGAG Reverse: TTGGTGGTTTGTGAGTGTGAG	Mouse
*Gapdh*	Forward: AGGTCGGTGTGAACGGATTTG Reverse: TGTAGACCATGTAGTTGAGGTCA	Mouse

**Table 3:** The sequences of target siRNAs in this study.

Gene	Sequences	Species
p62 siRNA	5’-GCUGAAACAUGGACACUUUTT-3’ 3’-AAAGUGUCCAUGUUUCAGCTT-5’	Mouse
RNF182 siRNA	5’-GCGCCAAAUGCCUCUACAATT-3’ 3’-UUGUAGAGGCAUUUGGCGCTT-5’	Mouse
RNF182 siRNA	5’-GACAACAACAUCCUUGUAATT-3’ 3’-UUACAAGGAUGUUGUUGUCTT-5’	Human
ING4 siRNA	5’-GAUCCCAACGAACCCACAUTT-3’ 3’-AUGUGGGUUCGUUGGGAUCTT-5’	Mouse
PPARγ siRNA	5’-GCAAGAGAUCACAGAGUAUTT-3’ 3’-AUACUCUGUGAUCUCUUGCTT-5’	Mouse

## Data Availability

The data presented in this study are available on request from the corresponding author.

## Supplementary Materials

The following supporting information can be downloaded at: https://www.mdpi.com/article/10.3390/nu14081673/s1, Figure S1: FR inhibits NF-κB signaling in tenocytes. (A) Tenocytes were treated with FR (0-40 μM) for 24 h, and then cell viability was detected by CCK8 assay. Tenocytes were treated with FR (0-40 μM) combined with LPS (100 ng/ml) or TNF-α (100 ng/ml) for 24 h, and then the cellular RNA, protein, and cell supernatant were collected for the following experiments. (B and C) The mRNA expression of IL-6 was detected by qRT-PCR. (D and E) The expression of IL-6 protein in cell supernatant was detected by ELISA. (F and H) The expression of NF-κB pathway-related proteins (p-IKK, IκBα, p-p65, and p65) were detected by WB. (G) Tenocytes were treated with FR (40 μM) and LPS (100 ng/ml) for 0-120 min, and then the expression of NF-κB pathway-related proteins (p-IKK, IκBα, p-p65, and p65) were detected by WB. The data are representative of three independent experiments. Error bars show the means ± SD. * *p* < 0.05, ** *p* < 0.01, *** *p* < 0.001. Figure S2. Silencing efficiency of siRNA. Tenocytes were transfected with RNF182 siRNA, ING4 siRNA, and PPARγ siRNA for 24 h, and the proteins expression of RNF182 (A), ING4 (B), and PPARγ (C) were detected by WB.
