# Supplementary material for: Friedelin Alleviates the Pathogenesis of Collagenase-Induced Tendinopathy in Mice by Promoting the Selective Autophagic Degradation of p65
Source: Nutrients. 2022 Apr 18;14(8):1673. doi: 10.3390/nu14081673 (PMC9031956; doi:10.3390/nu14081673)
Supplement: Supplementary file 1 [file nutrients-14-01673-s001.zip › nutrients-1614958-supplementary.pdf]

## Supplementary Materials

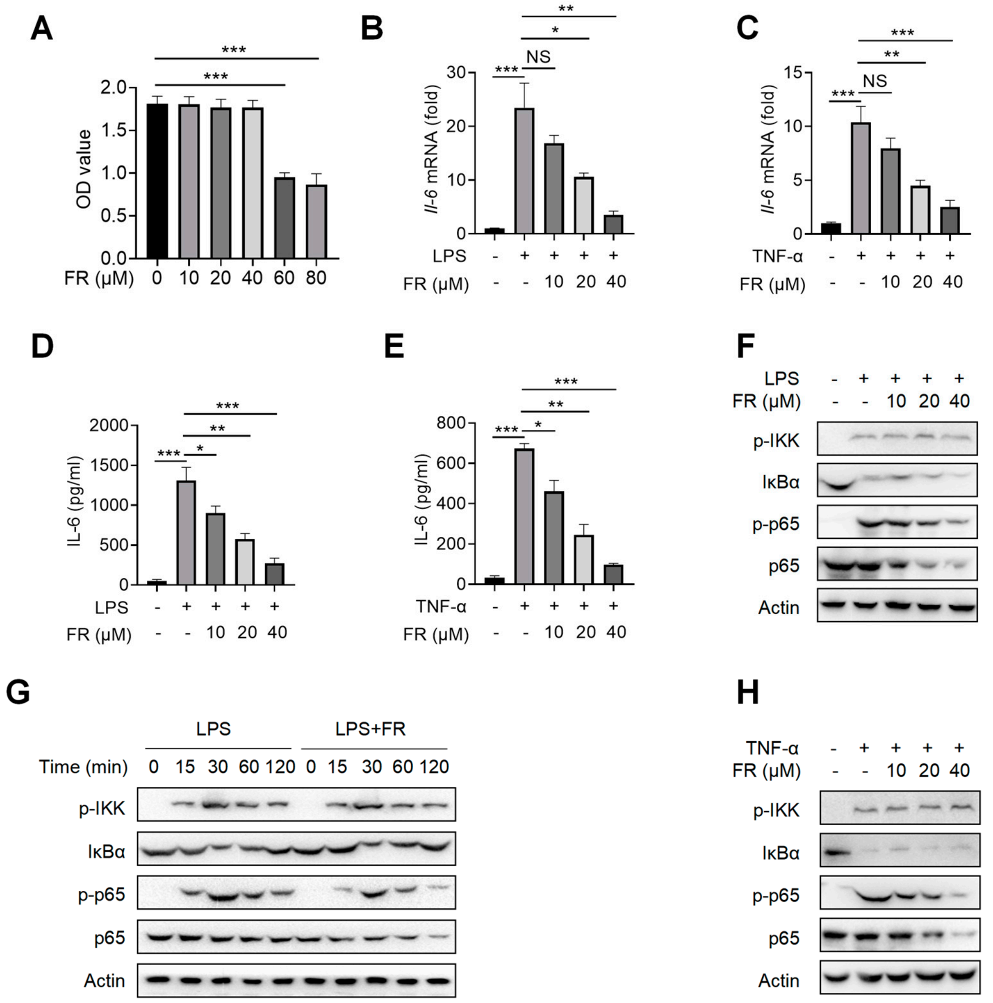


**Figure S1.** FR inhibits NF-κB signaling in tenocytes. **(A)**Tenocytes were treated with FR (0-40 μM) for 24 h, and then cell viability was detected by CCK8 assay. Tenocytes were treated with FR (0-40 μM) combined with LPS (100 ng/ml) or TNF-*α* (100 ng/ml) for 24 h, and then the cellular RNA, protein, and cell supernatant were collected for the following experiments. (**B** and **C**) The mRNA expression of IL-6 was detected by qRT-PCR. (**D** and **E**) The expression of IL-6 protein in cell supernatant was detected by ELISA. (**F** and **H**) The expression of NF-κB pathway-related proteins (p-IKK, IκB*α*, p-p65, and p65) were detected by WB. (**G**) Tenocytes were treated with FR (40 μM) and LPS (100 ng/ml) for 0-120 min, and then the expression of NF-κB pathway-related proteins (p-IKK, IκB*α*, p-p65, and p65) were detected by WB. The data are representative of three independent experiments. Error bars show the means ± SD. $^{*}P<0.05$, $^{**}P<0.01$, $^{***}P<0.001$.

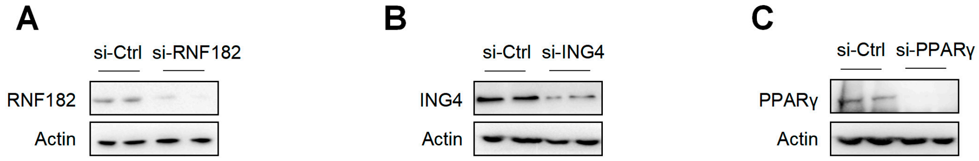


**Figure S2.** Silencing efficiency of siRNA.Tenocytes were transfected with RNF182 siRNA, ING4 siRNA, and PPARγ siRNA for 24 h, and the proteins expression of RNF182 (**A**), ING4 (**B**), and PPARγ (**C**) were detected by WB.
